# Artificial Intelligence in Medicine: Today and Tomorrow

**DOI:** 10.3389/fmed.2020.00027

**Published:** 2020-02-05

**Authors:** Giovanni Briganti, Olivier Le Moine

**Affiliations:** ^1^Medical Informatics, School of Medicine, Université Libre de Bruxelles, Brussels, Belgium; ^2^Unit of Epidemiology, Biostatistics and Clinical Research, School of Public Health, Université Libre de Bruxelles, Brussels, Belgium; ^3^Hopital Erasme, Université Libre de Bruxelles, Brussels, Belgium

**Keywords:** digital medicine, mobile health, medical technologies, artificial intelligence, monitoring

## Abstract

Artificial intelligence-powered medical technologies are rapidly evolving into applicable solutions for clinical practice. Deep learning algorithms can deal with increasing amounts of data provided by wearables, smartphones, and other mobile monitoring sensors in different areas of medicine. Currently, only very specific settings in clinical practice benefit from the application of artificial intelligence, such as the detection of atrial fibrillation, epilepsy seizures, and hypoglycemia, or the diagnosis of disease based on histopathological examination or medical imaging. The implementation of augmented medicine is long-awaited by patients because it allows for a greater autonomy and a more personalized treatment, however, it is met with resistance from physicians which were not prepared for such an evolution of clinical practice. This phenomenon also creates the need to validate these modern tools with traditional clinical trials, debate the educational upgrade of the medical curriculum in light of digital medicine as well as ethical consideration of the ongoing connected monitoring. The aim of this paper is to discuss recent scientific literature and provide a perspective on the benefits, future opportunities and risks of established artificial intelligence applications in clinical practice on physicians, healthcare institutions, medical education, and bioethics.

## 1. Introduction

The expression “Medical Technology” is widely used to address a range of tools that can enable health professionals to provide patients and society with a better quality of life by performing early diagnosis, reducing complications, optimizing treatment and/or providing less invasive options, and reducing the length of hospitalization. While, before the mobile era, medical technologies were mainly known as classic medical devices (e.g., prosthetics, stents, implants), the emergence of smartphones, wearables, sensors, and communication systems has revolutionized medicine with the capability of containing artificial intelligence (AI) powered tools (such as applications) in very small sizes (1). AI has revolutionized medical technologies and can be commonly understood as the part of computer science that is able to deal with complex problems with many applications in areas with huge amount of data but little theory (2).

Intelligent medical technologies (i.e., AI-powered) have been met with enthusiasm by the general population partly because it enables a 4P model of medicine (Predictive, Preventive, Personalized, and Participatory) and therefore patient autonomy, in ways that could not be possible (3); smartphones are becoming for instance the go-to item to fill and distribute an electronic personal health record (4), monitor vital functions with biosensors (5) and helping to reach optimal therapeutic compliance (6), therefore gifting the patient with the spot as the main actor in the care pathway. The development of intelligent medical technologies is enabling the development of a new field in medicine: augmented medicine, i.e., the use of new medical technologies to improve different aspects of clinical practice. Several AI-based algorithms have been approved in the last decade by the Food and Drug Administration (FDA) and could therefore be implemented. Augmented medicine is not only enabled by AI-based technologies but also several other digital tools, such as surgical navigation systems for computer-assisted surgery (7), virtuality-reality continuum tools for surgery, pain management and psychiatric disorders (8–10).

Although the field of augmented medicine seems to encounter success with patients, it can be met with a certain resistance by healthcare professionals, in particular physicians: concerning this phenomenon, four widely discussed reasons should be provided. First, unpreparedness as to the potential of digital medicine is due to the evident lack of basic and continuing education regarding this discipline (11). Second, the early digitization of healthcare processes, very different from the promise of augmented medicine came with a steep increase of the administrative burden mainly linked to electronic health records (12), which has come to be known as one of the main components of physician burnout (13). Third, there is increasing fear as to the risk of AI replacing physicians (14), although the current and mainstream opinion in the literature is that AI will complement physician intelligence in the future (15, 16). Fourth, the current world-wide lack of a legal framework that defines the concept of liability in the case of adoption or rejection of algorithm recommendations leaves the physician exposed to potential legal outcomes when using AI (17).

As of the lack of education in digital medicine, several private medical schools are preparing their future medical leaders to the challenge of augmented medicine by either associating the medical curriculum with the engineering curriculum or implementing digital health literacy and use in an upgraded curriculum (18).

The aim of this paper is to summarize recent developments of AI in medicine, provide the main use-cases where AI-powered medical technologies can already be used in clinical practice, and perspectives on the challenges and risks that healthcare professionals and institutions face while implementing augmented medicine, both in clinical practice and in the education of future medical leaders.

## 2. Current Applications of Artificial Intelligence in Medicine

### 2.1. Cardiology

#### 2.1.1. Atrial Fibrillation

The early detection of atrial fibrillation was one of the first application of AI in medicine. AliveCor received FDA approval in 2014 for their mobile application Kardia allowing for a smartphone-based ECG monitoring and detection of atrial fibrillation. The recent REHEARSE-AF study (19) showed that remote ECG monitoring with Kardia in ambulatory patients is more likely to identify atrial fibrillation than routine care. Apple also obtained FDA approval for their Apple Watch 4 that allows for easy acquirement of ECG and detection of atrial fibrillation that can be shared with the practitioner of choice through a smartphone (20). Several critiques of wearable and portable ECG technologies have been addressed (21), highlighting limitations to their use, such as the false positive rate originated from movement artifacts, and barriers in the adoption of wearable technology in the elderly patients that are more likely to suffer from atrial fibrillation.

#### 2.1.2. Cardiovascular Risk

Applied to electronic patient records, AI has been used to predict the risk of cardiovascular disease, for instance acute coronary syndrome (22) and heart failure (23) better than traditional scales. Recent comprehensive reviews (24) have however reported how results can vary depending on the sample size used in research report.

### 2.2. Pulmonary Medicine

The interpretation of pulmonary function tests has been reported as a promising field for the development of AI applications in pulmonary medicine. A recent study (25) reported how AI-based software provides more accurate interpretation and serves as a decision support tool in the case on interpreting results from pulmonary function tests. The study received several critiques, one of which (26) reported how the rate of accurate diagnosis in the pulmonologists participating in the study was considerably lower than the country average.

### 2.3. Endocrinology

Continuous glucose monitoring enables patients with diabetes to view real-time interstitial glucose readings and provides information on the direction and rate of change of blood glucose levels (27) Medtronic received FDA approval for their Guardian system for glucose monitoring, which is smartphone-paired (28). In 2018, the company partnered with Watson (AI developed by IBM) for their Sugar.IQ system to help their customers better prevent hypoglycemic episodes based on repeated measurement. Continuous blood glucose monitoring can enable patients to optimize their blood glucose control and reduce stigma associated with hypoglycemic episodes; however, a study focusing on patient experience with glucose monitoring reported that participants, while expressing confidence in the notifications, also declared feelings of personal failure to regulate glucose level (27).

### 2.4. Nephrology

Artificial intelligence has been applied in several settings in clinical nephrology. For instance, it has been proven useful for the prediction of the decline of glomerular filtration rate in patients with polycystic kidney disease (29), and for establishing risk for progressive IgA nephropathy (30). However, a recent review reporters how at this moment research is limited by sample size necessary for inference (31).

### 2.5. Gastroenterology

The specialty of gastroenterology benefits from wide range of AI applications in clinical settings. Gastroenterologists made use of convolutional neural networks among other deep learning models in order to process images from endoscopy and ultrasound (32) and detect abnormal structures such as colonic polyps (33). Artificial neural networks have also been used to diagnose gastroesophageal reflux disease (34) and atrophic gastritis (35), as well as to predict outcomes in gastrointestinal bleeding (36), survival of esophageal cancer (37), inflammatory bowel disease (38), and metastasis in colorectal cancer (39) and esophageal squamous cell carcinoma (40).

### 2.6. Neurology

#### 2.6.1. Epilepsy

Intelligent seizure detection devices are promising technologies that have the potential to improve seizure management through permanent ambulatory monitoring. Empatica received FDA approval in 2018 for their wearable Embrace, which associated with electrodermal captors can detect generalized epilepsy seizures and report to a mobile application that is able to alert close relatives and trusted physician with complementary information about patient localization (41). A report focused on patient experience, revealed that, in contrast to heart monitoring wearables, patients suffering from epilepsy had no barriers in the adoption of seizure detection devices, and reported high interest in wearable usage (42).

#### 2.6.2. Gait, Posture, and Tremor Assessment

Wearable sensors have proven useful to quantitatively assess gait, posture, and tremor in patients with multiple sclerosis, Parkinson disease, Parkinsonism, and Huntington disease (43).

### 2.7. Computational Diagnosis of Cancer in Histopathology

Paige.ai has received breakthrough status from FDA for an AI-based algorithm that is capable of diagnose cancer in computational histopathology with great accuracy, allowing pathologist to gain time to focus on important slides (44).

### 2.8. Medical Imaging and Validation of AI-Based Technologies

A long-awaited meta-analysis compared performances of deep learning software and radiologists in the field of imaging-based diagnosis (45): although deep learning seems to be as efficient as radiologist for diagnosis, the authors pointed that 99% of studies were found not to have a reliable design; furthermore, only one thousandth of the papers that were reviewed validated their results by having algorithms diagnose medical imaging coming from other source populations. These findings support the need of an extensive validation of AI-based technologies through rigorous clinical trials (5).

## 3. Discussion: Challenges and Future Directions of Artificial Intelligence in Medicine

### 3.1. Validation of AI-Based Technologies: Toward a Replication Crisis?

One of the core challenges of the application of AI in medicine in the next years will be the clinical validation of the core concepts and tools recently developed. Although many studies have already introduced the utility of AI with clear opportunities based on promising results, several well recognized and frequently reported limitations of AI studies are likely to complicate such validation. We will hereby address three of such limitations, as well as provide possible ways to overcome them.

First, the majority of studies comparing efficiency of AI vs. clinicians are found to have unreliable design and known to lack primary replication, i.e., the validation of the algorithms developed in samples coming from other sources than the one used to train algorithms (45). This difficulty could be overcome in the open science era as open data and open methods are bound to receive more and more attention as best practices in research. However, transitioning to open science could prove difficult for medical AI companies that develop software as a core business.

Second, studies reporting AI application in clinical practice are known to be limited because of retrospective designs and sample sizes; such designs potentially include selection and spectrum bias, i.e., models are developed to optimally fit a given data set (this phenomenon is also known as overfitting), but do not replicate the same results in other datasets (32). Continuous reevaluation and calibration after the adoption of algorithms that are suspected of overfitting should be necessary to adapt software to the fluctuation of patient demographics (46). Furthermore, there is a growing consensus as of the need of development of algorithms designed to fit larger communities while taking into account subgroups (47).

Third, only few studies are known to compare AI and clinicians based on same data sets; even in that scenario, critiques have been made pointing at lower diagnostic accuracy rate than expected in specialty doctors. (26). Opposing AI and clinicians is, although well represented in the scientific literature, probably not the best way to tackle the issue of performance in medical expertise: several studies are now approaching the interaction between clinicians and algorithms (47) as the combination of human and artificial intelligence outperforms either alone.

### 3.2. Ethical Implications of Ongoing Monitoring

Medical technology is one of the most promising markets of the 21st century, with an estimated market value rapidly approaching a thousand billion dollars in 2019. An increasing percentage of the revenue is due to the retail of medical devices (such as heart monitoring devices) to a younger population, which is not the primary target consumer profile (because health problems such as atrial fibrillation are less likely to appear). Because of this phenomenon, the Internet of Things (IoT) is redefining the concept of healthy individual as a combination of the quantified self (personal indicators coded in the smartphone or wearable) and series of lifestyle wearable-provided parameters (activity monitoring, weight control, etc.).

Furthermore, in the last couple of years several wearable companies have been concluding important deals with either insurance companies or governments to organize a large-scale distribution of these products: this kind of initiatives are mainly aimed to induce lifestyle change in large populations. While western countries are continuing to evolve toward health systems centered around the patient's individual responsibility toward its own health and well-being, the ethical implications of ongoing medical monitoring with medical devices through the Internet of things are frequently discussed. For instance, ongoing monitoring and privacy violations have the potential to increase stigma around chronically ill or more disadvantaged citizens (48) and possibly penalize those citizens that are unable to adopt new standards of healthy lifestyle, for instance by reducing access to health insurance and care; little to no debate has been focused on these potential and crucial pitfalls in health policy making.

In this techno-political framework, the issue of data protection and ownership becomes more and more crucial, although more than two decades old (49). Several attitudes toward data ownership are described in the literature: although some works argue for common ownership of patients data to profit personalized medicine approaches (50, 51), consensus is shifting toward patient ownership, as it has positive effects on patient engagement as well as may improve information sharing if a data use agreement between the patient and healthcare professionals is developed (52).

### 3.3. The Need to Educate Augmented Doctors

Several universities have started to create new medical curriculum, including a doctor-engineering (18), to answer the need of educating future medical leaders to the challenges of artificial intelligence in medicine (53). Such curricula see a stronger approach to the hard sciences (such as physics and mathematics), and the addition of computational sciences, coding, algorithmics, and mechatronic engineering. These “augmented doctors” would count on both a clinical experience and digital expertise to solve modern health problems, participate in defining digital strategies for healthcare institutions, manage the digital transition, educate patients and peers.

Society as well as healthcare institutions could benefit from these professionals as a safety net for any processes including AI in medicine but also as a drive of innovation and research. Aside from basic medical education, there is a need for implementation of ongoing educational programs regarding digital medicine and targeting graduated physicians, so as to allow retraining in this growing field. In most cutting-edge hospitals around the world, such experts are charged with the mission of Chief Medical Information Officer (CMIO).

### 3.4. The Promise of Ambient Clinical Intelligence: Avoiding Dehumanization by Technology

As reported by several studies (12, 13), electronic health records can be an important administrative burden and a source of burnout, phenomenon increasingly present in physicians, both in training and trained. Although artificial intelligence solutions such as Natural Language Processing are becoming more and more capable of helping the physician deliver complete medical records, further solutions are needed to solve the issue of the increasing time allocated to indirect patient care.

Ambient clinical intelligence (ACI) is understood as a sensitive, adaptive and responsive digital environment surrounding the physician and the patient (54) and capable of, for instance, analyzing the interview and automatically fill the patient's electronic health records. Several projects are underway to develop an ACI, which would be a crucial application of artificial intelligence in medicine and much needed to solve modern problems with the physician workforce.

One of the great barriers to the adoption of intelligent medical technologies in physicians is the fear of a dehumanization of medicine. This is mainly due to the increasing administrative burden (12) imposed on physicians. However, modern technology such as ACI and Natural Language processing are bound to solve the issue of administrative burden and will help clinicians focus more on the patient.

### 3.5. Will Doctors Be Replaced by Artificial Intelligence?

As recently discussed in the literature (15, 16) doctors will most likely not be replaced by artificial intelligence: smart medical technologies exist as such as support to the physician in order to improve patient management. As recent studies have indicated (45), however, comparisons frequently occur between artificial intelligence solutions and physicians, as if the two counterparts were in competition. Future studies should focus on the comparison between physicians using artificial intelligence solutions with physicians without the aid of such applications, and extend those comparisons to translational clinical trials; only then will artificial intelligence be accepted as complementary to physicians. Healthcare professionals stand nowadays in a privileged position, to be able to welcome the digital evolution and be the main drivers of change, although a major revision of medical education is needed to provide future leaders with the competences to do so.

## 4. Conclusion

The implementation of artificial intelligence in clinical practice is a promising area of development, that rapidly evolves together with the other modern fields of precision medicine, genomics and teleconsultation. While scientific progress should remain rigorous and transparent in developing new solutions to improve modern healthcare, health policies should now be focused on tackling the ethical and financial issues associated with this cornerstone of the evolution of medicine.

## Author Contributions

All authors listed have made a substantial, direct and intellectual contribution to the work, and approved it for publication.

### Conflict of Interest

The authors declare that the research was conducted in the absence of any commercial or financial relationships that could be construed as a potential conflict of interest.
